# 2,3-Diamino­pyridinium 2-hy­droxy­benzoate

**DOI:** 10.1107/S1600536811044461

**Published:** 2011-10-29

**Authors:** Madhukar Hemamalini, Jia Hao Goh, Hoong-Kun Fun

**Affiliations:** a X-ray Crystallography Unit, School of Physics, Universiti Sains Malaysia, 11800 USM, Penang, Malaysia

## Abstract

In the title mol­ecular salt, C_5_H_8_N_3_^+^·C_7_H_5_O_3_^−^, the 2,3-diamino­pyridinium cation is essentially planar, with a maximum deviation of 0.006 (2) Å. In the crystal, adjacent cations and anions are linked by pairs of N—H⋯O hydrogen bonds, generating *R*_2_^2^(8) loops. These dimers are linked by further N—H⋯O hydrogen bonds and C—H⋯O inter­actions to form sheets lying parallel to (001). A typical intra­molecular O—H⋯O hydrogen bond is also observed in the salicylate (2-hy­droxy­benzoate) anion, which generates an *S*(6) ring. The crystal structure also features π–π stacking inter­actions between the pyridinium rings of the cations, with a centroid–centroid distance of 3.5896 (15) Å.

## Related literature

For details of 2-amino­pyridine and its derivatives, see: Banerjee & Murugavel (2004 ▶); Bis & Zaworotko (2005 ▶); Bis *et al.* (2006 ▶). For hydrogen-bond motifs, see: Bernstein *et al.* (1995 ▶). For bond-length data, see: Allen *et al.* (1987 ▶). For the stability of the temperature controller used in the data collection, see: Cosier & Glazer (1986 ▶).
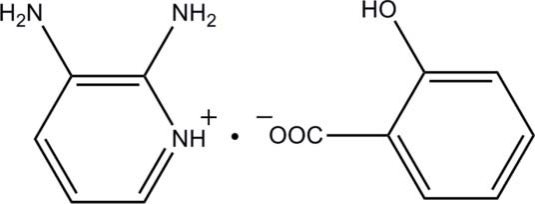


## Experimental

### 

#### Crystal data

C_5_H_8_N_3_^+^·C_7_H_5_O_3_^−^
*M* *_r_* = 247.25
Orthorhombic, 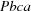

*a* = 10.484 (3) Å
*b* = 11.260 (3) Å
*c* = 20.033 (6) Å
*V* = 2364.9 (12) Å^3^
*Z* = 8
Mo *K*α radiation
μ = 0.10 mm^−1^
*T* = 100 K
0.54 × 0.47 × 0.10 mm

#### Data collection

Bruker APEXII DUO CCD diffractometer
Absorption correction: multi-scan (*SADABS*; Bruker, 2009 ▶) *T*_min_ = 0.947, *T*_max_ = 0.990
12688 measured reflections
3388 independent reflections
1989 reflections with *I* > 2σ(*I*)
*R*_int_ = 0.063

#### Refinement

*R*[*F* ^2^ > 2σ(*F* ^2^)] = 0.050
*wR*(*F* ^2^) = 0.144
*S* = 1.03
3388 reflections
216 parameters
All H-atom parameters refined
Δρ_max_ = 0.21 e Å^−3^
Δρ_min_ = −0.21 e Å^−3^

### 

Data collection: *APEX2* (Bruker, 2009 ▶); cell refinement: *SAINT* (Bruker, 2009 ▶); data reduction: *SAINT*; program(s) used to solve structure: *SHELXTL* (Sheldrick, 2008 ▶); program(s) used to refine structure: *SHELXTL*; molecular graphics: *SHELXTL*; software used to prepare material for publication: *SHELXTL* and *PLATON* (Spek, 2009 ▶).

## Supplementary Material

Crystal structure: contains datablock(s) global, I. DOI: 10.1107/S1600536811044461/hb6455sup1.cif

Structure factors: contains datablock(s) I. DOI: 10.1107/S1600536811044461/hb6455Isup2.hkl

Supplementary material file. DOI: 10.1107/S1600536811044461/hb6455Isup3.cml

Additional supplementary materials:  crystallographic information; 3D view; checkCIF report

## Figures and Tables

**Table 1:** Hydrogen-bond geometry (Å, °)

*D*—H⋯*A*	*D*—H	H⋯*A*	*D*⋯*A*	*D*—H⋯*A*
O1—H1*O*1⋯O3	0.96 (2)	1.69 (2)	2.573 (2)	151.2 (18)
N1—H1*N*1⋯O2	0.94 (2)	1.80 (2)	2.736 (2)	171.1 (19)
N2—H1*N*2⋯O3	0.900 (19)	2.019 (19)	2.905 (2)	167.7 (18)
N2—H2*N*2⋯O2^i^	0.91 (2)	2.04 (2)	2.942 (2)	169.6 (18)
N3—H1*N*3⋯O2^i^	0.92 (2)	1.99 (2)	2.907 (2)	174.3 (19)
N3—H2*N*3⋯O3^ii^	0.88 (2)	2.14 (2)	2.994 (2)	163.9 (19)
C7—H7⋯O3^iii^	0.99 (2)	2.56 (2)	3.376 (3)	139.8 (17)

Symmetry codes: (i) 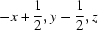; (ii) 
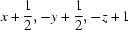
; (iii) 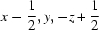.

**Fig. 1.**
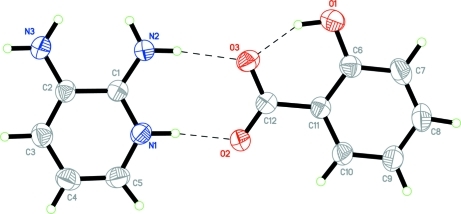
The asymmetric unit of the title compound, showing 50% probability displacement ellipsoids. Hydrogen bonds are indicated by dashed lines.

**Fig. 2.**
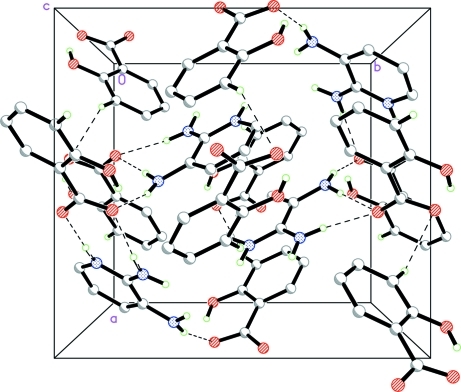
The crystal packing of title compound (I).

## supplementary crystallographic information

### Comment

2-Aminopyridine and its derivatives are some of the most frequently used synthons in supramolecular chemistry based on hydrogen bonds (Banerjee & Murugavel, 2004; Bis & Zaworotko, 2005; Bis *et al.*, 2006). In this paper, we report the crystal structure of the title compound, (I), which belongs to this class of compounds.

The asymmetric unit, (Fig 1), contains a protonated 2,3-diaminopyridinium cation and a benzoate anion. The 2,3-diaminopyridinium cation is planar, with a maximum deviation of 0.006 (2) Å for atom C1.  The diheral angle between the pyridine (N1/C1–C5) and the benzene (C6–C11) rings is 3.35 (9)°.  The bond lengths (Allen *et al.*, 1987) and angles are normal. A typical intramolecular O—H···O hydrogen bond is also observed in the salicylate anion, which generates an *S*(6) ring.

In the crystal packing, the protonated N1 atom and the 2-amino group (N2) is hydrogen-bonded to the carboxylate oxygen atoms (O2 and O3) *via* a pair of N—H···O hydrogen bonds forming an *R*^2^_2_(8) ring motif (Bernstein *et al.*, 1995). The cationic and anionic units are linked through N—H···O and C—H···O hydrogen bonds (Table 1 and Fig 2) to form a two-dimensional network parallel to the (0 0 1) plane. The crystal structure is further stabilized by π–π stacking interactions between the pyridinium (Cg1 = N1/C1–C5) rings (Cg1···Cg1 = 3.5896 (15) Å; 1-x, 1-y, 1-z) of the cations.

### Experimental

Hot methanol solutions (20 ml) of 2,3-diaminopyridine (27 mg, Aldrich) and salicylic acid ( 34 mg, Merck) were mixed and warmed over a heating magnetic stirrer for 5 minutes. The resulting solution was allowed to cool slowly at room temperature.  Brown plates of the title compound appeared from the mother liquor after a few days.

### Refinement

All hydrogen atoms were located from a difference Fourier maps and refined freely [N–H = 0.88 (2)–0.94 (2) Å; O–H = 0.96 (2) Å and C–H = 0.93 (2)–1.01 (2) Å].

### Crystal data

**Table:**

C_5_H_8_N_3_^+^·C_7_H_5_O_3_^−^	*F*(000) = 1040
*M**_r_* = 247.25	*D*_x_ = 1.389 Mg m^−^^3^
Orthorhombic, *P**b**c**a*	Mo *K*α radiation, λ = 0.71073 Å
Hall symbol: -P 2ac 2ab	Cell parameters from 2044 reflections
*a* = 10.484 (3) Å	θ = 2.8–27.3°
*b* = 11.260 (3) Å	µ = 0.10 mm^−^^1^
*c* = 20.033 (6) Å	*T* = 100 K
*V* = 2364.9 (12) Å^3^	Plate, brown
*Z* = 8	0.54 × 0.47 × 0.10 mm

### Data collection

**Table:**

Bruker APEXII DUO CCD diffractometer	3388 independent reflections
Radiation source: fine-focus sealed tube	1989 reflections with *I* > 2σ(*I*)
graphite	*R*_int_ = 0.063
φ and ω scans	θ_max_ = 30.0°, θ_min_ = 2.8°
Absorption correction: multi-scan (*SADABS*; Bruker, 2009)	*h* = −14→14
*T*_min_ = 0.947, *T*_max_ = 0.990	*k* = −8→15
12688 measured reflections	*l* = −27→23

### Refinement

**Table:**

Refinement on *F*^2^	Secondary atom site location: difference Fourier map
Least-squares matrix: full	Hydrogen site location: inferred from neighbouring sites
*R*[*F*^2^ > 2σ(*F*^2^)] = 0.050	All H-atom parameters refined
*wR*(*F*^2^) = 0.144	*w* = 1/[σ^2^(*F*_o_^2^) + (0.0573*P*)^2^ + 0.2876*P*] where *P* = (*F*_o_^2^ + 2*F*_c_^2^)/3
*S* = 1.03	(Δ/σ)_max_ < 0.001
3388 reflections	Δρ_max_ = 0.21 e Å^−^^3^
216 parameters	Δρ_min_ = −0.21 e Å^−^^3^
0 restraints	Extinction correction: *SHELXTL* (Sheldrick, 2008), Fc^*^=kFc[1+0.001xFc^2^λ^3^/sin(2θ)]^-1/4^
Primary atom site location: structure-invariant direct methods	Extinction coefficient: 0.0084 (15)

### Special details

**Table:**

Experimental. The crystal was placed in the cold stream of an Oxford Cryosystems Cobra open-flow nitrogen cryostat (Cosier & Glazer, 1986) operating at 100.0 (1) K.
Geometry. All esds (except the esd in the dihedral angle between two l.s. planes) are estimated using the full covariance matrix. The cell esds are taken into account individually in the estimation of esds in distances, angles and torsion angles; correlations between esds in cell parameters are only used when they are defined by crystal symmetry. An approximate (isotropic) treatment of cell esds is used for estimating esds involving l.s. planes.
Refinement. Refinement of F^2^ against ALL reflections. The weighted R-factor wR and goodness of fit S are based on F^2^, conventional R-factors R are based on F, with F set to zero for negative F^2^. The threshold expression of F^2^ > 2sigma(F^2^) is used only for calculating R-factors(gt) etc. and is not relevant to the choice of reflections for refinement. R-factors based on F^2^ are statistically about twice as large as those based on F, and R- factors based on ALL data will be even larger.

### Fractional atomic coordinates and isotropic or equivalent isotropic displacement parameters (Å^2^)

**Table:**

	*x*	*y*	*z*	*U*_iso_*/*U*_eq_	
N1	0.28118 (14)	0.47769 (11)	0.49919 (8)	0.0372 (3)	
N2	0.31269 (15)	0.31738 (13)	0.42951 (8)	0.0399 (4)	
N3	0.49319 (18)	0.22674 (14)	0.52153 (10)	0.0515 (4)	
C1	0.34064 (15)	0.37422 (13)	0.48633 (8)	0.0329 (4)	
C2	0.43007 (15)	0.33158 (13)	0.53385 (9)	0.0356 (4)	
C3	0.45227 (17)	0.39879 (16)	0.58971 (10)	0.0427 (4)	
C4	0.38911 (19)	0.50631 (16)	0.60051 (11)	0.0462 (5)	
C5	0.30414 (18)	0.54408 (15)	0.55483 (10)	0.0441 (4)	
O1	−0.04088 (13)	0.39481 (11)	0.24975 (8)	0.0517 (4)	
O2	0.09892 (12)	0.57050 (10)	0.41657 (6)	0.0432 (3)	
O3	0.08969 (11)	0.40353 (9)	0.35839 (6)	0.0392 (3)	
C6	−0.09061 (16)	0.50000 (14)	0.26928 (9)	0.0377 (4)	
C7	−0.18426 (18)	0.54995 (18)	0.22901 (11)	0.0494 (5)	
C8	−0.23979 (19)	0.65666 (18)	0.24675 (11)	0.0502 (5)	
C9	−0.20179 (18)	0.71566 (17)	0.30334 (11)	0.0475 (5)	
C10	−0.10732 (17)	0.66749 (14)	0.34298 (10)	0.0418 (4)	
C11	−0.04980 (14)	0.55892 (13)	0.32692 (9)	0.0331 (4)	
C12	0.05192 (15)	0.50816 (13)	0.37001 (8)	0.0331 (4)	
H3	0.5167 (19)	0.3712 (17)	0.6237 (10)	0.056 (6)*	
H4	0.4035 (18)	0.5488 (16)	0.6398 (10)	0.049 (5)*	
H5	0.2538 (19)	0.6171 (18)	0.5580 (10)	0.054 (5)*	
H7	−0.212 (2)	0.5068 (18)	0.1886 (12)	0.066 (6)*	
H8	−0.304 (2)	0.6964 (16)	0.2196 (10)	0.050 (5)*	
H9	−0.2398 (17)	0.7915 (16)	0.3162 (9)	0.042 (5)*	
H10	−0.0825 (19)	0.7010 (16)	0.3859 (11)	0.048 (5)*	
H1O1	0.016 (2)	0.3733 (17)	0.2853 (11)	0.059 (6)*	
H1N1	0.224 (2)	0.5073 (16)	0.4672 (10)	0.048 (5)*	
H1N2	0.2509 (18)	0.3436 (15)	0.4020 (10)	0.038 (5)*	
H2N2	0.3459 (18)	0.2443 (19)	0.4211 (10)	0.054 (6)*	
H1N3	0.465 (2)	0.1816 (17)	0.4864 (11)	0.056 (6)*	
H2N3	0.536 (2)	0.1958 (16)	0.5553 (11)	0.052 (6)*	

### Atomic displacement parameters (Å^2^)

**Table:**

	*U*^11^	*U*^22^	*U*^33^	*U*^12^	*U*^13^	*U*^23^
N1	0.0357 (8)	0.0335 (7)	0.0423 (9)	0.0034 (6)	−0.0022 (7)	−0.0001 (6)
N2	0.0419 (8)	0.0376 (7)	0.0401 (9)	0.0062 (7)	−0.0096 (7)	−0.0050 (6)
N3	0.0599 (11)	0.0414 (8)	0.0531 (11)	0.0154 (8)	−0.0212 (9)	−0.0047 (8)
C1	0.0295 (8)	0.0312 (7)	0.0380 (9)	−0.0034 (6)	−0.0004 (7)	0.0020 (6)
C2	0.0347 (8)	0.0330 (7)	0.0391 (10)	−0.0017 (7)	−0.0035 (7)	0.0037 (7)
C3	0.0405 (10)	0.0447 (9)	0.0429 (11)	−0.0018 (8)	−0.0079 (8)	0.0017 (8)
C4	0.0495 (11)	0.0454 (9)	0.0436 (11)	−0.0020 (9)	−0.0021 (9)	−0.0086 (8)
C5	0.0453 (10)	0.0373 (8)	0.0496 (12)	0.0035 (8)	−0.0010 (9)	−0.0071 (8)
O1	0.0476 (8)	0.0510 (8)	0.0565 (9)	0.0040 (6)	−0.0077 (7)	−0.0173 (6)
O2	0.0458 (7)	0.0389 (6)	0.0449 (8)	0.0057 (5)	−0.0135 (6)	−0.0061 (5)
O3	0.0405 (6)	0.0319 (6)	0.0453 (7)	0.0023 (5)	0.0005 (5)	−0.0002 (5)
C6	0.0315 (8)	0.0394 (8)	0.0423 (10)	−0.0046 (7)	0.0007 (7)	−0.0031 (7)
C7	0.0397 (10)	0.0614 (12)	0.0471 (12)	−0.0056 (9)	−0.0103 (9)	−0.0050 (9)
C8	0.0377 (10)	0.0561 (11)	0.0568 (13)	0.0006 (9)	−0.0133 (9)	0.0056 (9)
C9	0.0405 (10)	0.0403 (9)	0.0617 (13)	0.0022 (8)	−0.0126 (9)	0.0024 (9)
C10	0.0380 (9)	0.0362 (8)	0.0513 (12)	0.0001 (7)	−0.0092 (8)	−0.0021 (8)
C11	0.0276 (8)	0.0321 (7)	0.0395 (10)	−0.0052 (6)	−0.0004 (7)	0.0019 (6)
C12	0.0304 (8)	0.0324 (7)	0.0366 (9)	−0.0028 (6)	0.0031 (7)	0.0032 (7)

### Geometric parameters (Å, °)

**Table:**

N1—C1	1.346 (2)	O1—C6	1.352 (2)
N1—C5	1.364 (2)	O1—H1O1	0.96 (2)
N1—H1N1	0.94 (2)	O2—C12	1.267 (2)
N2—C1	1.338 (2)	O3—C12	1.2646 (19)
N2—H1N2	0.90 (2)	C6—C7	1.390 (3)
N2—H2N2	0.91 (2)	C6—C11	1.399 (2)
N3—C2	1.376 (2)	C7—C8	1.382 (3)
N3—H1N3	0.92 (2)	C7—H7	0.99 (2)
N3—H2N3	0.88 (2)	C8—C9	1.373 (3)
C1—C2	1.420 (2)	C8—H8	0.97 (2)
C2—C3	1.371 (3)	C9—C10	1.380 (3)
C3—C4	1.397 (3)	C9—H9	0.977 (18)
C3—H3	1.01 (2)	C10—C11	1.401 (2)
C4—C5	1.346 (3)	C10—H10	0.97 (2)
C4—H4	0.93 (2)	C11—C12	1.486 (2)
C5—H5	0.98 (2)		
			
C1—N1—C5	123.31 (15)	N1—C5—H5	114.7 (12)
C1—N1—H1N1	118.2 (12)	C6—O1—H1O1	104.2 (12)
C5—N1—H1N1	118.4 (12)	O1—C6—C7	117.32 (16)
C1—N2—H1N2	121.4 (12)	O1—C6—C11	122.45 (16)
C1—N2—H2N2	120.4 (13)	C7—C6—C11	120.22 (16)
H1N2—N2—H2N2	117.3 (17)	C8—C7—C6	120.02 (18)
C2—N3—H1N3	117.2 (13)	C8—C7—H7	120.9 (12)
C2—N3—H2N3	116.5 (13)	C6—C7—H7	119.1 (12)
H1N3—N3—H2N3	122.2 (18)	C9—C8—C7	120.72 (19)
N2—C1—N1	118.37 (15)	C9—C8—H8	116.0 (11)
N2—C1—C2	123.58 (15)	C7—C8—H8	123.2 (11)
N1—C1—C2	118.05 (15)	C8—C9—C10	119.54 (18)
C3—C2—N3	122.58 (16)	C8—C9—H9	121.5 (11)
C3—C2—C1	118.21 (15)	C10—C9—H9	118.9 (11)
N3—C2—C1	119.18 (16)	C9—C10—C11	121.31 (17)
C2—C3—C4	121.63 (17)	C9—C10—H10	123.2 (11)
C2—C3—H3	119.6 (11)	C11—C10—H10	115.2 (11)
C4—C3—H3	118.7 (11)	C6—C11—C10	118.16 (15)
C5—C4—C3	118.84 (18)	C6—C11—C12	121.10 (14)
C5—C4—H4	121.2 (12)	C10—C11—C12	120.74 (15)
C3—C4—H4	119.9 (12)	O3—C12—O2	122.00 (15)
C4—C5—N1	119.95 (17)	O3—C12—C11	118.43 (14)
C4—C5—H5	125.3 (12)	O2—C12—C11	119.57 (14)
			
C5—N1—C1—N2	−178.69 (16)	C6—C7—C8—C9	1.3 (3)
C5—N1—C1—C2	1.1 (2)	C7—C8—C9—C10	−0.2 (3)
N2—C1—C2—C3	178.90 (16)	C8—C9—C10—C11	−0.5 (3)
N1—C1—C2—C3	−0.9 (2)	O1—C6—C11—C10	−179.94 (16)
N2—C1—C2—N3	0.5 (3)	C7—C6—C11—C10	1.0 (2)
N1—C1—C2—N3	−179.27 (15)	O1—C6—C11—C12	0.2 (2)
N3—C2—C3—C4	178.57 (18)	C7—C6—C11—C12	−178.86 (16)
C1—C2—C3—C4	0.2 (3)	C9—C10—C11—C6	0.0 (3)
C2—C3—C4—C5	0.2 (3)	C9—C10—C11—C12	179.93 (16)
C3—C4—C5—N1	−0.1 (3)	C6—C11—C12—O3	−8.9 (2)
C1—N1—C5—C4	−0.6 (3)	C10—C11—C12—O3	171.22 (15)
O1—C6—C7—C8	179.24 (17)	C6—C11—C12—O2	170.49 (15)
C11—C6—C7—C8	−1.7 (3)	C10—C11—C12—O2	−9.4 (2)

### Hydrogen-bond geometry (Å, °)

**Table:**

*D*—H···*A*	*D*—H	H···*A*	*D*···*A*	*D*—H···*A*
O1—H1O1···O3	0.96 (2)	1.69 (2)	2.573 (2)	151.2 (18)
N1—H1N1···O2	0.94 (2)	1.80 (2)	2.736 (2)	171.1 (19)
N2—H1N2···O3	0.900 (19)	2.019 (19)	2.905 (2)	167.7 (18)
N2—H2N2···O2^i^	0.91 (2)	2.04 (2)	2.942 (2)	169.6 (18)
N3—H1N3···O2^i^	0.92 (2)	1.99 (2)	2.907 (2)	174.3 (19)
N3—H2N3···O3^ii^	0.88 (2)	2.14 (2)	2.994 (2)	163.9 (19)
C7—H7···O3^iii^	0.99 (2)	2.56 (2)	3.376 (3)	139.8 (17)

Symmetry codes: (i) −*x*+1/2, *y*−1/2, *z*; (ii) *x*+1/2, −*y*+1/2, −*z*+1; (iii) *x*−1/2, *y*, −*z*+1/2.

## References

[bb1] Allen, F. H., Kennard, O., Watson, D. G., Brammer, L., Orpen, A. G. & Taylor, R. (1987). *J. Chem. Soc. Perkin Trans. 2*, pp. S1–19.

[bb2] Banerjee, S. & Murugavel, R. (2004). *Cryst. Growth Des.* **4**, 545–552.

[bb3] Bernstein, J., Davis, R. E., Shimoni, L. & Chang, N.-L. (1995). *Angew. Chem. Int. Ed. Engl.* **34**, 1555–1573.

[bb4] Bis, J. A., McLaughlin, O. L., Vishweshwar, P. & Zaworotko, M. J. (2006). *Cryst. Growth Des.* **6**, 2648–2650.

[bb5] Bis, J. A. & Zaworotko, M. A. (2005). *Cryst. Growth Des.* **5**, 1169–1179.

[bb6] Bruker (2009). *APEX2*, *SAINT* and *SADABS* Bruker AXS Inc., Madison, Wisconsin, USA.

[bb7] Cosier, J. & Glazer, A. M. (1986). *J. Appl. Cryst.* **19**, 105–107.

[bb8] Sheldrick, G. M. (2008). *Acta Cryst.* A**64**, 112–122. 10.1107/S0108767307043930 18156677

[bb9] Spek, A. L. (2009). *Acta Cryst.* D**65**, 148–155. 10.1107/S090744490804362X PMC2631630 19171970

